# Potential application of mixed metal oxide nanoparticle-embedded glassy carbon electrode as a selective 1,4-dioxane chemical sensor probe by an electrochemical approach

**DOI:** 10.1039/c9ra09118a

**Published:** 2019-12-18

**Authors:** Mohammed M. Rahman, M. M. Alam, Abdullah M. Asiri

**Affiliations:** Chemistry Department, Faculty of Science, King Abdulaziz University Jeddah 21589 P. O. Box 80203 Saudi Arabia alam-mahmud@hotmail.com; Department of Chemical Engineering and Polymer Science, Shahjalal University of Science and Technology Sylhet 3100 Bangladesh

## Abstract

Here, low-dimensional mixed metal oxide (ZnO/NiO/MnO_2_) nanoparticles (NPs) were prepared to develop a selective, efficient and ultra-sensitive 1,4-dioxane sensor by using the wet-chemical method (co-precipitation) in alkaline medium at low temperature. Detailed characterization of the prepared calcined NPs was achieved *via* conventional methods, including X-ray diffraction, field emission scanning electron microscopy, and X-ray photoelectron, UV-vis, Fourier-transform infrared and energy dispersive X-ray spectroscopies. To develop a thin layer of nanomaterial on the fabricated electrode, a slurry of prepared NPs was used to coat the glassy carbon electrode (GCE) with conductive Nafion (5% in ethanol) binder. The fabricated electrochemical sensor showed good sensitivity (1.0417 μA μM^−1^ cm^−2^), a wide linear dynamic range (0.12 nM to 1.2 mM), lower detection limit (9.14 ± 4.55 pM), short response time, good reproducibility, and long-term stability to selectively detect 1,4-dioxane in the optimized buffer system. Thus, this work presents a reliable alternative approach over existing methods to selectively detect hazardous chemicals in large scale for safety in the environmental and healthcare fields.

## Abstract 2

Low-dimensional ternary ZnO/NiO/MnO_2_ nanoparticles were prepared by wet-chemical co-precipitation in alkaline medium and then used to develop a selective and ultra-sensitive 1,4-dioxane sensor using electrochemistry for the safety of healthcare and the environment.

## Graphical abstract

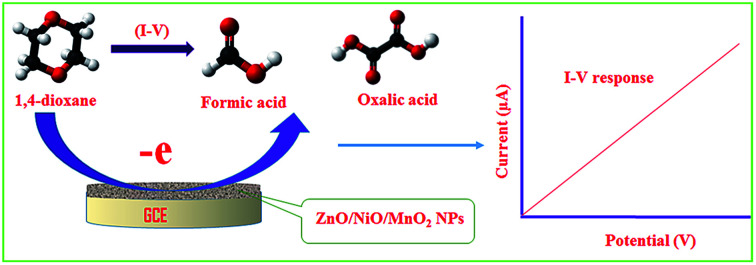

## Introduction

The cyclic diether 1,4-dioxane is a well-known nonbiodegradable organic compound that is used as solvent in various industrial processes, including the production of paints, inks, dyes, varnish, pharmaceuticals, paper, fabric cleaners and electronics.^1,2^ Also, it is used extensively to produce consumer and personal care items. Besides this, 1,4-dioxane is obtained as a by-product during the manufacture of ethylene oxide and ethylene glycol.^3^ However, the excessive industrial application and high solubility of 1,4-dioxane has led to the pollution of ground and surface water.^4,5^ Therefore, 1,4-dioxane contamination of water is found to be significantly increased in the industrial regions of the world.^6–8^ Due to the high toxicity of 1,4-dioxane, these industrial effluents are a great threat to humans and environmental ecosystems.^9^ Therefore, much research has been done on the detection of 1,4-dioxane, a possible carcinogen. The International Agency for Research on Cancer (IARC) has listed 1,4-dioxane as a probable human carcinogen.^10,11^ Furthermore, it is suspected that 1,4-dioxane is also responsible for damage to the nervous system and failure of kidney, lungs, and liver in humans.^12,13^ To increase public awareness, a trustworthy detection method for 1,4-dioxane is essential. To date, a number of methods have been applied to detect 1,4-dioxane, such as high-performance liquid chromatography (HPLC),^14^ solid phase extraction-gas chromatography/mass spectrometry (SPE-GC/MS),^15,16^ and thermal desorption (TD) gas chromatography (GC).^17^ However, these methods are limited in the trace detection of 1,4-dioxane. Therefore, a simple and efficient method for the detection of 1,4-dioxane in aqueous medium is required. Recently, the electrochemical approach has become prevalent due to its reliable and effective trace detection of toxic chemicals in aqueous media.^18,19^

Due to their excellent electrochemical properties, nanostructured semiconductors and transition metal oxides have been utilized as sensing elements in numerous chemical sensing applications.^20,21^ Among the transition metal oxides, a ZnO p-type semiconductor, with a band-gap energy of 3.3 eV, has been investigated to detect xanthine,^22^ 2-nitrophenol,^23^ 1,2-dichlorobenzene^24^ and hydrazine^25^ in aqueous media. Another p-type semi-conductor, NiO, with a band-gap energy of 4.0 eV, has been reported as efficient and reliable in sensor applications to detect 4-aminophenol^26^ and 4-methoxyphenol.^27^ Therefore, p-type semi-conductive nanostructured metal oxides with wide band-gap energies are suitable as sensing materials in the field of electrochemical sensor development. Furthermore, MnO_2_ is a promising semi-conductive metal oxide with low resistance, large specific surface area, and high catalytic activity, along with an attractive electrochemical response.^28^ Additionally, due to its promising optical–electrical properties, SnO_2_ composited with various metal and metal oxides has exhibited potential as a sensing element to detect gas.^29–31^ Thus, the approach of this research is to develop a consistent 1,4-dioxane chemical sensor using a ternary mixture of the semi-conductive nanostructured metal oxides ZnO, NiO, and MnO_2_.

In this study, an electrochemical sensor with ternary metal oxide ZnO/NiO/MnO_2_ nanoparticles on GCE was developed and applied to selectively detect 1,4-dioxane in aqueous medium by electrochemical method. The proposed 1,4-dioxane chemical sensor was found to exhibit higher sensitivity, broad linear dynamic range, short response time, lower detection limit, excellent reproducibility, and long-term stability.

## Experimental section

### Chemicals and reagents

The laboratory-grade chemicals Zn(NO_3_)_2_·6H_2_O, NiCl_2_·6H_2_O and MnSO_4_·H_2_O were obtained from Sigma-Aldrich Company and used directly as received to prepare the ternary semi-conductive metal oxide ZnO/NiO/MnO_2_ NPs. Toxic chemicals, such as zimtaldehyde, paracetamol, acetonitrile, xanthine, ethylene glycol, benzyl chloride, diethyl malonate, 1,4-dioxane, 3-methylaniline, and chlorobenzene were procured to execute this study.

### Synthesis of mixed metal oxide NPs

The preparation of the ZnO/NiO/MnO_2_ NPs in this study was found to be a sensitive task. A co-precipitation (wet-chemical method) technique was implemented to synthesize ZnO/NiO/MnO_2_ NPs in alkaline medium. Wet-chemical techniques, particularly co-precipitation, are the oldest methods used to synthesize metal oxides or composites. Presently, the solvothermal and sol–gel methods are extensively implemented to prepare various metal oxides and composites.^32–38^ To perform the wet-chemical (co-precipitation) process, 0.1 M solutions of Zn(NO_3_)_2_·6H_2_O, NiCl_2_·6H_2_O and MnSO_4_·H_2_O were prepared in three individual 100 mL round-bottom flasks using deionized water. Then, 50.0 mL of each prepared solution was poured into a 250 mL conical flask and allowed to heat at 85.0 °C using a hot plate with continuous magnetic stirring. To gradually increase the pH of the solution to 10.5, an ammonium hydroxide solution of 0.1 M in concentration was added dropwise, and all the metal ions were co-precipitated in the form of metal hydroxides [Zn(OH)_2_, Ni(OH)_2_ and Mn(OH)_2_]. Under these conditions, the conical flask was kept for several hours on the hot plate with magnetic stirring. The proposed reaction scheme in aqueous (alkaline) medium is presented below.

i NH_4_OH_(l)_ ⇆ NH_4(aq)_^+^ + OH_(aq)_^−^

ii Zn(NO_3_)_2(s)_ → Zn_(aq)_^2+^ + 2NO_3(aq)_^−^

iii MnSO_4(s)_ → Mn_(aq)_^2+^ + 2SO_4(aq)_^−^

iv NiCl_2(s)_ → Ni_(aq)_^2+^ + 2Cl_(aq)_^−^

v Zn_(aq)_^2+^ + Mn_(aq)_^2+^ + Ni_(aq)_^2+^ + OH_(aq)_^−^ + *n*H_2_O ⇆ Zn(OH)_2_·*x*Mn(OH)_2_·*x*Ni(OH)_2(s)_·*n*H_2_O↓

In the wet-chemical method, precipitation of metal hydroxides depends on the value of the solubility product constant, *K*_s_. The reported *K*_s_ values are 3 × 10^−27^, 5.48 × 10^−16^, and 1.9 × 10^−13^, corresponding to Zn(OH)_2_, Ni(OH)_2_ and Mn(OH)_2_, in alkaline pH medium.^39^ Due to the dropwise addition of NH_4_OH solution, the pH of the reaction medium slowly increases. Since Zn(OH)_2_ has a lower *K*_s_ value, it starts to precipitate first and forms the nuclei of crystal formation. Then, the resulting crystallites aggregate to form larger crystallites. With increasing pH value, Ni(OH)_2_ starts to precipitate and attach to crystallites of Zn(OH)_2._ Similarly, the last semi-conductive metal hydroxide (Mn(OH)_2_) is precipitated out. Thus, at pH 10.5, all metal ions are precipitated out quantitatively. The obtained metal hydroxides were separated from the aqueous medium and consequently washed with acetone and deionized water, successively. Next, the precipitate was placed inside a low-temperature oven and allowed to dry at 110.0 °C overnight. To obtain the metal oxide from the metal hydroxide, the resultant dry sample was subjected to calcination in a high-temperature muffle furnace at 500.0 °C for 5 h. In this process, all the metal hydroxides are transformed to their oxide forms with higher oxidation number in the presence of atmospheric O_2_. The corresponding reaction inside the muffle furnace is given below.

vi Zn(OH)_2_·*x*Mn(OH)_2_·*x*Ni(OH)_2(s)_ + O_2_ → ZnO·*x*NiO·*x*MnO_2_ + H_2_O_(v)_

### Instruments

The following instruments and methods were used to complete the characterization, electrode preparation, and sensor application of the NPs. To investigate the photosensitivity of the calcined ZnO/NiO/MnO_2_ NPs, a 300 UV/visible spectrophotometer (Thermo Scientific) was implemented to measure the UV-vis spectra, and a NICOLET iS50 FTIR spectrometer (Madison, WI, USA) was used in the range of 400–4000 cm^−1^ to identify the functional groups. The oxidation states and binding energies of species were measured by X-ray photoelectron spectroscopic (XPS) analysis using a Kα1 spectrometer (Thermo Scientific, Kα 1066). Beside this, the morphology of the prepared ZnO/NiO/MnO_2_ NPs was inspected by field emission scanning electron microscopy (FESEM, JEOL, JSM-7600, Japan), and the respective elemental analysis was executed by energy dispersive X-ray spectroscopy (EDS, JEOL, Japan). Moreover, the crystallinity of synthesized NPs was analyzed by X-ray diffraction. Electrochemical investigations were carried out using an electrometer (651-Keithley Electrometer, USA).

### Fabrication of GCE electrode with ZnO/NiO/MnO_2_ NPs

The dominant part of this electrochemical sensor is the working electrode. To prepare such working electrode, a glassy carbon electrode (GCE) with a surface area of 0.0316 cm^2^ was coated with a slurry of ZnO/NiO/MnO_2_ NPs and dried under ambient conditions. Then, a drop of Nafion (5% in ethanol) was added, and the modified GCE was dried inside an oven at 35.0 °C for an hour to completely dry the thin film of ZnO/NiO/MnO_2_ NPs. Nafion is a co-polymer, and it acts as a conducting binder. As a result, the fabricated electrode shows long-term stability in aqueous medium as well as improved electron transfer rate of the electrode.^40,41^ To accomplish electrochemical analysis *via* an electrochemical method, an electrochemical sensor was assembled with a Keithley Electrometer, ZnO/NiO/MnO_2_ NPs/binder/GCE and Pt-wire. Several 1,4-dioxane solutions with varying concentrations ranging from 0.012 M to 0.12 nM were prepared and used as analyte in the assembled electrochemical sensor. The slope of the calibration curve, which resulted from plotting the concentration of 1,4-dioxane *vs.* the current, was used to measure the sensitivity, detection limit (DL) and linear dynamic range (LDR) of the proposed electrochemical sensor. During electrochemical measurements, the buffer solution in the inspecting beaker was kept constant at 10.0 mL for the entire experiment.

## Results and discussion

### Morphological and elemental analysis of ZnO/NiO/MnO_2_ NPs

**Fig. 1:**
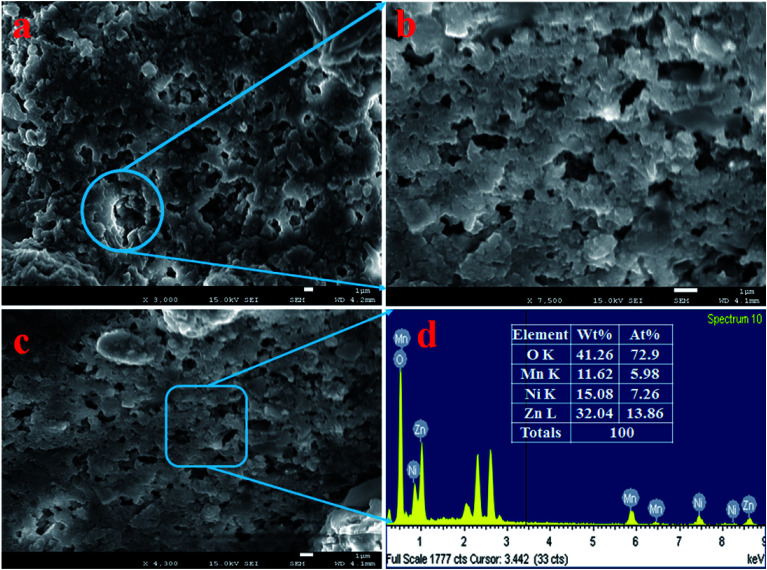
Evaluation of structural morphology and composition of the elements present in the synthesized NPs. (a and b) Low and high magnification FESEM images of ZnO/NiO/MnO_2_ NPs, and (c and d) elemental composition of NPs analyzed by EDS.

The morphology of ZnO/NiO/MnO_2_ nanoparticles was examined and is presented in Fig. 1(a and b). As observed from Fig. 1, the low and high magnification FESEM show the aggregation of irregularly shaped NPs, which are mesoporous in nature. Thus, it is concluded that the synthesized nanomaterials formed particles, as identified earlier.^42,43^ To confirm the elemental composition of the prepared ZnO/NiO/MnO_2_ NPs, EDS analysis was executed, as illustrated in Fig. 1(c and d). From the pattern shown in Fig. 1(d), the prepared NPs consist of Zn, Ni, Mn, and O only. The analyzed elemental compositions are 32.04%, 15.08%, 11.62%, and 41.26%, corresponding to Zn, Ni, Mn, and O.

### Binding energy and oxidation states

**Fig. 2:**
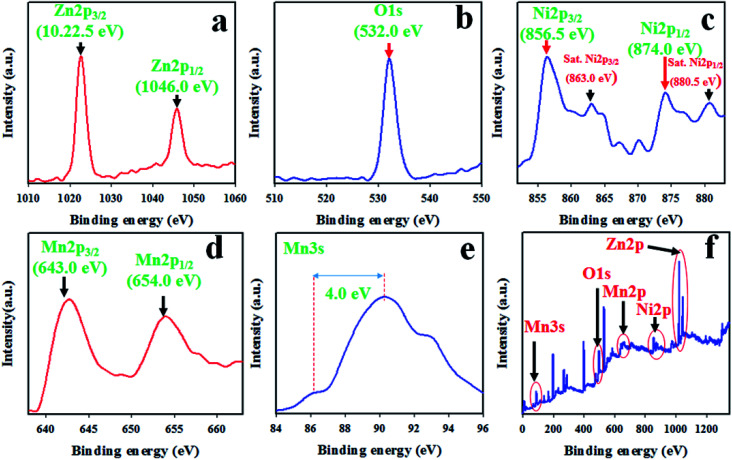
The binding energy and oxidation states of ZnO/NiO/MnO_2_ NPs, quantified by XPS analysis: (a) core-level spin orbit of Zn 2p, (b) XPS spectrum of O 1s level orbit, (c) spin orbit of Ni 2p level, (d) Mn 2p spin orbit, (e) spin orbit of Mn 3s level, and (f) XPS survey spectrum of ZnO/NiO/MnO_2_ NPs.

XPS was performed for the detailed study of the binding energy and oxidation states of ZnO/NiO/MnO_2_ NPs prepared using the wet-chemical technique. The resulting XPS spectra are presented in Fig. 2. The core level spectrum of Zn 2p is presented in Fig. 2(a), and it shows two identical peaks at 1022.5 and 1046.0 eV, corresponding to the Zn 2p_3/2_ and Zn 2p_1/2_ spin orbits, respectively, with a 23.5 eV spin–orbit splitting. This observation has been reported as Zn^2+^ in ZnO.^44–47^ As shown in Fig. 2(b), the O 1s XPS peak is positioned at 532.0 eV and is associated with the absorption of –OH group on the surface of ZnO/NiO/MnO_2_ NPs.^48–52^ Moreover, the XPS spectrum of Ni 2p is displayed in Fig. 2(c), in which the two main XPS peaks of Ni 2p are situated at 856.5 and 874.0 eV, associated with Ni 2p_3/2_ and Ni 2p_1/2_ spin-orbits, respectively, and the spin energy separation between these two peaks is equal to 17.5 eV. This value fits with the oxidation of Ni^2+^ in NiO. Furthermore, the core level spectrum of Ni 2p shows two satellite peaks located at 863.0 and 880.5 eV, related to Ni 2p_3/2_ and Ni 2p_1/2_ with spin energy separation of 17.5 eV. Therefore, both the main and satellite peaks of Ni 2p are associated with equal oxidation of Ni^2+^ in NiO.^53–56^ According to Fig. 2(d), the Mn 2p orbits show two peaks at 643.0 and 654.0 eV, matching to Mn 2p_3/2_ and Mn 2p_1/2_ individually. The spin energy separation between these two peaks is 11.0 eV. However, it is really difficult to distinguish between Mn^2+^ and Mn^3+^ oxidation states. To confirm the oxidation level of Mn 2p, the XPS spectrum of Mn 3s was explored, and as demonstrated in Fig. 2(e), the Mn 3s spin–orbits show a separation of 4.0 eV. Therefore, the Mn in the composite has a charge state of +4.0.^57–60^ From Fig. 2(f), the survey spectrum shows that the prepared NPs consist of Ni, Zn, O, and Mn only.

### Optical and phase crystallinity analyses

**Fig. 3:**
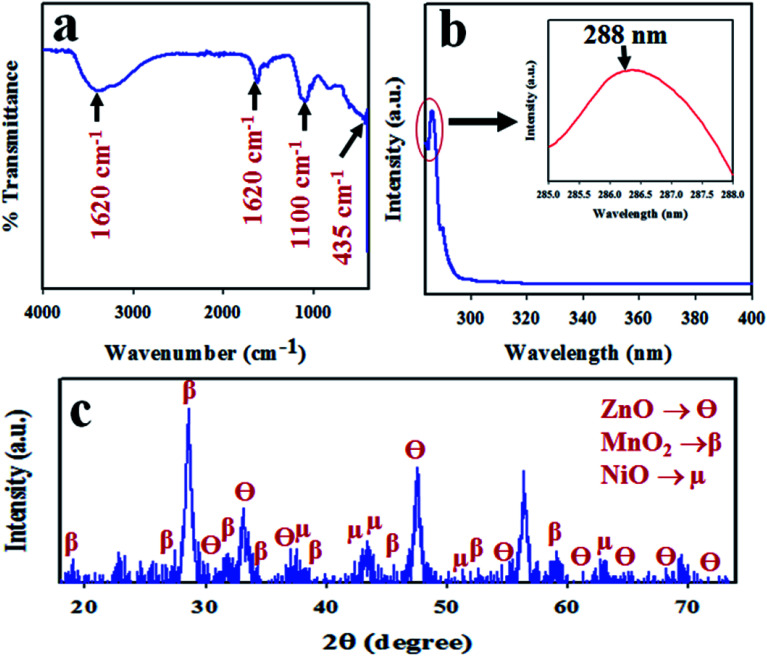
Analyses of the optical properties and crystallinity of the ZnO/NiO/MnO_2_ NPs: (a) FTIR spectrum, (b) UV-vis spectrum, and (c) XRD spectrum.

To confirm the functional groups present in the synthesized ZnO/NiO/MnO_2_ NPs, FTIR investigation was executed. The resultant FTIR spectrum is illustrated in Fig. 3(a); it demonstrates absorption bands at 435, 1100, 1620, and 3400 cm^−1^. The broad and intense absorption band at 435 cm^−1^ is associated with the stretching vibration mode of Zn–O or Mn–O.^61–63^ The resultant absorption band at 1100 cm^−1^ resembles the coordination of Mn by O–H bond,^64,65^ as seen in Fig. 3(a). Furthermore, the absorption bands corresponding to the O–H stretching vibration and H–O–H bending vibration due to the adsorption of water molecule are, respectively, 3400 and 1620 cm^−1^.^66–71^ The photosensitivity of ZnO/NiO/MnO_2_ NPs was evaluated by the implementation of UV-vis analysis, shown in Fig. 3(b). As seen in the UV-vis spectrum, an absorption band appears at 288 nm, which is the characteristic absorption band for ZnO/NiO/MnO_2_ NPs. A similar observation has been reported for ZnO–NiO–Fe_2_O_3_ and Cd-doped MnO_2_.^71,72^ The band-gap energy has been calculated from the highest point in the absorption band of ZnO/NiO/MnO_2_ NPs following eqn (vii), and the estimated band-gap energy is equal to 4.31 eV.

vii *E*_bg_ (eV) = 1240/*λ*

Here, *E*_bg_ is band-gap energy, and *λ* is the maximum absorption wavelength.

To analyse the phase crystallinity, powder X-ray diffraction analysis was carried out on the synthesized ZnO/NiO/MnO_2_ NPs, with the applied range of 2*θ* = 16–76°. The resultant XRD pattern is given in Fig. 3(c). A number of typical diffraction peaks of ZnO, designated as θ, are assigned to the (100), (002), (101), (102), (110), (103), (200), (112), (201), and (004) planes. A similar finding has been reported for ZnO by previous authors^73–75^ and matched with standard X-ray diffraction data (JCPDS 0005-0664). Beside this, the crystalline diffraction peaks for MnO_2_ indices, designated as β, are attributed to the planes (101), (112), (200), (103), (211), (004), (220), (105), and (224), which are analogous with JCPDS no. 0041-1442 and earlier reports.^76–78^ Moreover, the studied XRD shows some diffracted peaks of NiO directories, designated as μ, of (111), (200), and (220). This finding is supported by JCPDS no. 0047-1049 and other reports.^79,80^ The Scherer equation [eqn (vii)] is used to measure the crystalline particle size at the highest β peak of (200),^81^ and it is found to be 14.28 nm.

viii *D* = 0.9*λ*/(*β* cos *θ*)

Here, *λ* is the wavelength of X-ray radiation (1.5418 Å), and *β* is full width at half maximum (FWHM) of the peak at diffraction angle *θ*.

**Fig. 4:**
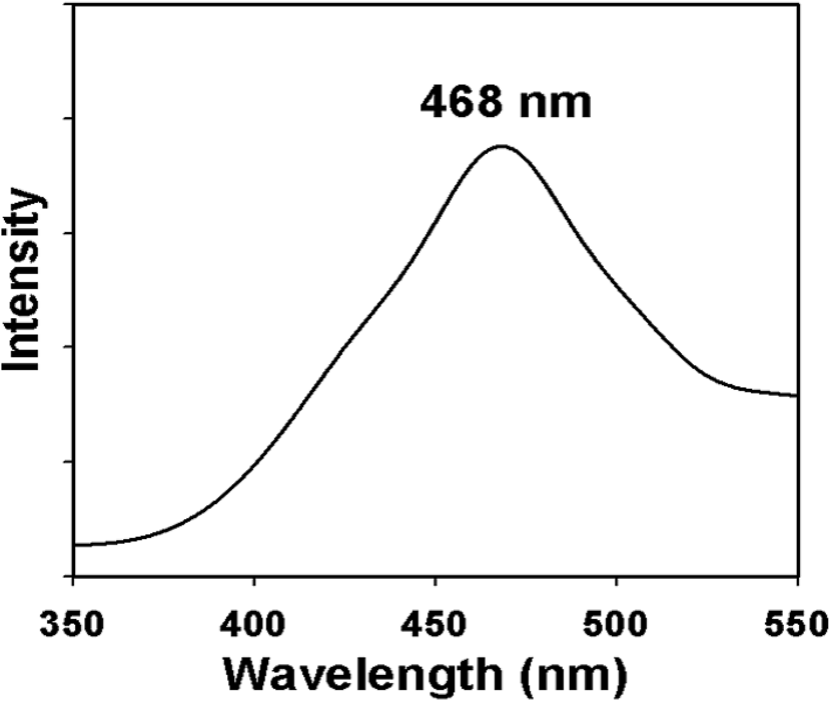
Emission spectrum (PL) of ternary mixed ZnO/NiO/MnO2 NPs.

Additionally, photoluminescence (PL) spectroscopy is a powerful tool to investigate the quality, purity and optical properties of the nanostructured materials. Owing to the excellent light absorption properties of nanomaterials, they might be very fascinating for further research in electrocatalytic as well as in photocatalytic devices. The PL emission spectrum of the synthesized ZnO/NiO/MnO_2_ NPs at excitation wavelength gives a peak in the visible region at 468 nm, corresponding to blue emission (Fig. 4). The emission band in the blue region is due to transition vacancies of oxygen and interstitial oxygen. The band corresponding to blue emission is also due to a singly ionized oxygen vacancy. This ZnO/NiO/MnO_2_ NPs show strong blue emission band in the visible region, which is in good consensus with the literature.^82,83^

### Optimization and evaluation of the analytical performance of the sensor

**Fig. 5:**
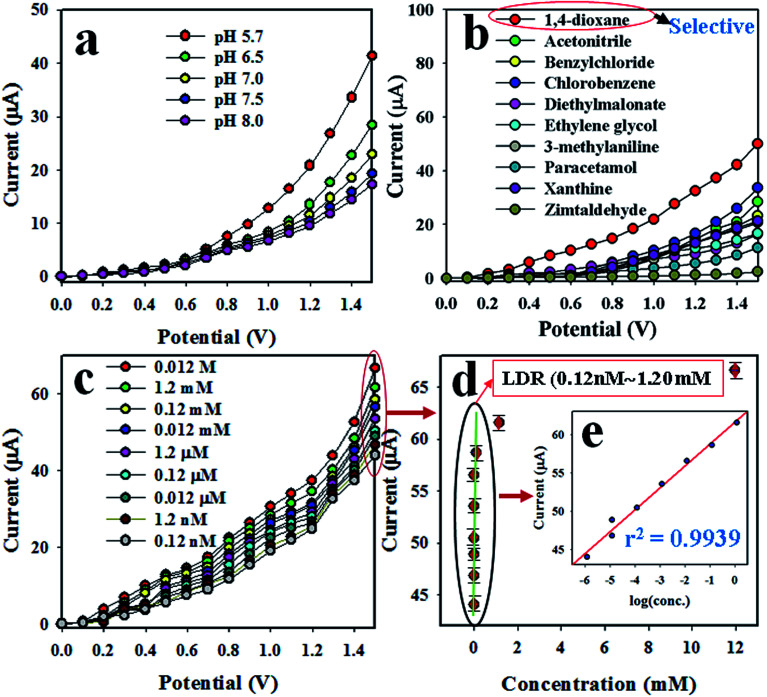
Optimization of 1,4-dioxane chemical sensor based on ZnO/NiO/MnO_2_ NPs/binder/GCE: (a) optimization of pH, (b) selectivity determination, (c) electrochemical responses of 1,4-dioxane from lower to higher concentration, (d) calibration plot [inset (e): log(conc.) *vs.* the current].

The desired chemical sensor was achieved using ZnO/NiO/MnO_2_ NPs coated as a thin layer on a GCE. The binding strength between the NPs and GCE was boosted by adding a drop of Nafion, a conducting binder. To confirm the appropriate activity of the ZnO/NiO/MnO_2_ NPs/binder/GCE sensor, several phosphate buffer media, with pH values ranging from 5.7 to 8.0, were tested, and the sensor's performance was examined at an applied potential of 0 to +1.5 V. As is apparent from Fig. 5(a), the electrochemical sensor gives the highest electrochemical response in a buffer with a pH value of 5.7. The selectivity of this predictable chemical sensor was assessed by the analysis of environmental toxic chemicals at micro-level concentrations and an applied potential of 0 to +1.5 V. The electrochemical responses towards zimtaldehyde, paracetamol, acetonitrile, xanthine, ethylene glycol, benzyl chloride, diethyl malonate, 1,4-dioxane, 3-methylaniline, and chlorobenzene are exemplified in Fig. 5(b), and perceptibly, 1,4-dioxane shows the maximum activity toward the probable ZnO/NiO/MnO_2_ NPs/binder/GCE chemical sensor in the optimized buffer system at pH 5.7. The analytical-grade 0.12 M 1,4-dioxane has been diluted in deionized water to prepare a number of solutions ranging in concentration from 0.012 M to 0.12 nM, and the corresponding electrochemical response of each 1,4-dioxane solution is plotted in Fig. 5(c). As seen in the figure, the electrochemical responses are distinct from lower to higher concentrations of 1,4-dioxane; this performance was measured at an applied potential of 0 to +1.5 V. To estimate the analytical parameters of the 1,4-dioxane chemical sensor, current data were collected from Fig. 5(c) at an applied potential of +1.5 V and plotted as the concentration of 1,4-dioxane *vs.* the current, as illustrated in Fig. 5(d), denoted as the calibration curve. The sensitivity of the anticipated 1,4-dioxane chemical sensor was assessed from the slope of the calibration curve and surface area of the GCE (0.0316 cm^2^), and was found to be 1.0417 μA μM^−1^ cm^−2^, a result that is realistically high. As observed from Fig. 5(d), the current data are homogeneously scattered along a linear plot over the concentration range of 0.12 nM to 1.2 mM 1,4-dioxane, giving a linear dynamic range (LDR) that evidences the reliability of our method. To explore the linearity of the calibration plot within the linear dynamic range (0.12 nM to 1.2 mM), the current data are plotted against concentration in logarithmic scale, as shown in Fig. 5(e). The current data are coordinated with a regression coefficient *r*^2^ = 0.9939. In short, it is apparent that the 1,4-dioxane chemical sensor is able to detect 1,4-dioxane in a real application. The detection limit of the 1,4-dioxane chemical sensor was determined from the slope of calibration curve using a signal-to-noise ratio of 3, and it is equal to 9.14 ± 4.55 pM, a value that is actually very low.

**Scheme 1:**
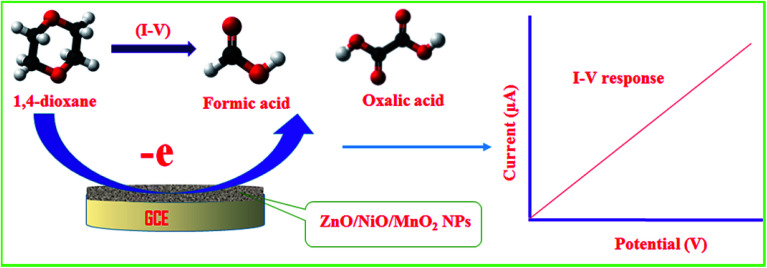
The proposed detection mechanism of 1,4-dioxane in aqueous medium.

As can be seen from Fig. 5(c), the electrochemical response of the 1,4-dioxane chemical sensor is amplified with a boost in 1,4-dioxane concentration in the sensing medium. A proposed pictorial representation of the 1,4-dioxane detection process is revealed in Scheme 1. The 1,4-dioxane itself is a Lewis base, and it is able to donate electrons at pH 5.7.^84,85^ Therefore, during the electrochemical sensing of 1,4-dioxane in an optimized buffer system with a pH of 5.7, 1,4-dioxane is oxidized to generate formic acid and oxalic acid. Simultaneously, the density of electrons in the sensing medium is found to be enhanced, which is responsible for the high conductivity of the sensing medium. As a result, the electrochemical responses vary with the concentration of 1,4-dioxane. A similar oxidation of 1,4-dioxane has been reported by previous authors.^86–90^

### Reliability and stability of the electrochemical method

**Fig. 6:**
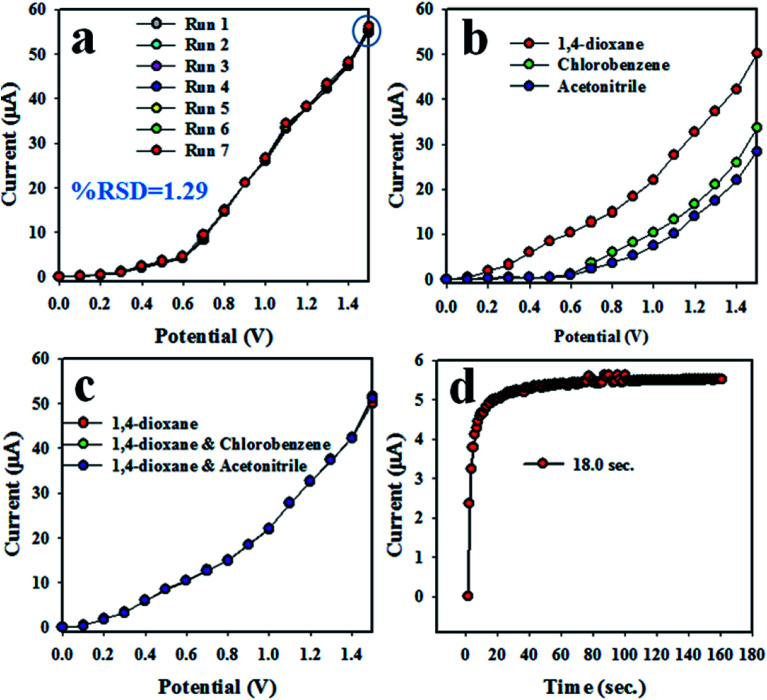
Reliability performance of 1,4-dioxane chemical sensor based on ZnO/NiO/MnO_2_ NPs/binder/GCE: (a) reproducibility of sensor, (b) comparison of *I*–*V* response with 1,4-dioxane and its mixtures with chlorobenzene and acetonitrile, respectively, (c) interference study of the 1,4-dioxane chemical sensor, and (d) response time.

The reproducibility of the chemical sensor is a key analytical parameter in sensor development. Therefore, the reproducibility of the anticipated 1,4-dioxane electrochemical sensor was tested at 0.012 μM 1,4-dioxane and the potential of 0 to +1.5 V, and the results are demonstrated in Fig. 6(a). As seen in Fig. 6(a), the seven replicated runs are basically indistinguishable, and the electrochemical responses are not altered, even with washing of the electrode after each trial. Thus, it can be settled that the proposed chemical will be consistent in real-field application to detect 1,4-dioxane in aqueous medium. To explore the precision of the 1,4-dioxane chemical sensor, the relative standard deviation (RSD) of the current data at an applied potential of +1.5 V was calculated, and the measured RSD is found to be 1.29%, a result showing little appreciable differences. The analogous reproducibility test of the 1,4-dioxane chemical sensor was performed over four days, and the findings are represented in Fig. 6(a). Therefore, from Fig. 6(a), it can be predicted that the 1,4-dioxane chemical sensor has long-term stability with consistency in performance, under identical conditions. Fig. 6(b) shows the comparison of electrochemical responses between 1,4-dioxane, chlorobenzene and acetonitrile. It can be observed that 1,4-dioxane demonstrates the highest electrochemical response compared to other toxins at 0.012 μM concentration. Fig. 6(c) shows the electrochemical responses towards 1,4-dioxane only, 1,4-dioxane & chlorobenzene, and 1,4-dioxane & acetonitrile. These electrochemical responses are completely indistinguishable. Thus, it can be supposed that the demonstrated 1,4-dioxane chemical sensor is selective to 1,4-dioxane, and no interference effect is found in the presence of other toxins. The response time is another indicator of the analytical performance of the chemical sensor. As presented in Fig. 6(d), the obtained response time for the 1,4-dioxane chemical sensor is around 18.0 s; this experiment was executed at 0.012 μM concentration of 1,4-dioxane solution. Therefore, considering the sensor analytical performance in terms of sensitivity, linear dynamic range (LDR), detection limit (DL), response time, reproducibility, and accuracy of the electrochemical responses, the 1,4-dioxane chemical sensor exhibits highly significant performance.

To investigate the validity of the study, a comparison with similar research using various nanocomposites or nanomaterials was explored^89,90^ and is presented in Table 1. The analytical parameters, such as sensitivity, LDR and DL, were found to be quite high and satisfactory.

**Table 1:** Comparison of the electrochemical sensing performance of electrodes based on various nanocomposite materials^a^

Modified GCE	DL	LDR	Sensitivity	Ref.
PANI-SiO_2_ NCs	16.0 pM	0.12 nM to 1.2 mM	0.5934 μA μM^−1^ cm^−2^	89
NiO@Nd_2_O_3_ NCs/GCE	33.0 pM	0.12 nM to 0.12 mM	0.029 μA μM^−1^ cm^−2^	90
ZnO/NiO/MnO_2_ NPs/GCE	9.14 pM	0.12 nM to 1.2 mM	1.0417 μA μM^−1^ cm^−2^	This study

a DL (detection limit), LDR (linear dynamic range), pM (picomolar), mM (millimolar).

### Application of sensor in real samples using the recovery method

The ultimate target of the development of this chemical sensor is its application in the real environmental field. Therefore, the anticipated 1,4-dioxane chemical sensor probe has been applied to analyze real environmental samples for the selective detection of 1,4-dioxane. The recovery method is a process in which a known concentration of the sensing target (*e.g.*, 1,4-dioxane) is added to a real water sample, and subsequently, the concentration is measured by the fabricated chemical sensor. Here, this detection process is executed by recovery method. The real samples have been obtained from various sources, including sea water (Red Sea, Jeddah, KSA), extracts from a PC-baby bottle, PVC-food packaging bag, and PVC-water bottle, and waste effluent from the Jeddah industrial zone. The analysis data are represented in Table 2 and appear to be quite satisfactory.

**Table 2:** Investigation of environmental samples using the ZnO/NiO/MnO_2_ NPs/binder/GCE sensor probe *via* a recovery method

Sample	Added 1,4-dioxane conc. (μM)	Measured 1,4-dioxane conc. by ZnO/NiO/MnO_2_ NPs/GCE^a^ (μM)			Average recovery^b^ (%)	RSD^c^ (%) (*n* = 3)
		R1	R2	R3		
Industrial effluent	0.012000	0.011949	0.011957	0.011752	99.05	0.98
PC-baby bottle	0.012000	0.011868	0.011901	0.011883	99.03	0.14
PVC-water bottle	0.012000	0.011985	0.011854	0.011772	98.92	0.91
PVC-food packaging bag	0.012000	0.011807	0.011789	0.011894	98.58	0.47
Sea water	0.012000	0.011765	0.011734	0.011761	97.94	0.14

a Mean of three repeated determinations (signal-to-noise ratio of 3) using ZnO/NiO/MnO_2_ NPs/GCE.

b Concentration of 1,4-dioxane determined/concentration taken (unit: μM).

c Relative standard deviation value indicates precision among three repeated measurements (R1, R2, R3).

## Conclusion

In this research, a selective 1,4-dioxane chemical sensor was developed based on wet-chemically synthesized low-dimensional mixed metal oxide (ZnO/NiO/MnO_2_) NPs coated onto a GCE sensor probe with 5% Nafion conducting binder. The prepared ZnO/NiO/MnO_2_ NPs were fully characterized using XRD, XPS, FESEM, EDS, FTIR and UV-vis analysis. The proposed 1,4-dioxane chemical sensor exhibits reliable and substantial performance, as revealed by considering its excellent sensing parameters, such as higher sensitivity, wide linear dynamic range, short response time, ultra-low detection limit, and reproducibility with high accuracy. Therefore, this research method might pave the way for the development of a selective chemical sensor for the large-scale detection of unsafe environmental contaminants in the environmental and healthcare fields.

## Conflicts of interest

There are no conflicts to declare.

## Supplementary Material
